# Supplementary material for: Metabolic Effects of New Glucose Transporter (GLUT-1) and Lactate Dehydrogenase-A (LDH-A) Inhibitors against Chemoresistant Malignant Mesothelioma
Source: Int J Mol Sci. 2023 Apr 24;24(9):7771. doi: 10.3390/ijms24097771 (PMC10177874; doi:10.3390/ijms24097771)
Supplement: Supplementary file 1 [file ijms-24-07771-s001.zip › ijms-2344209-supplementary.pdf]

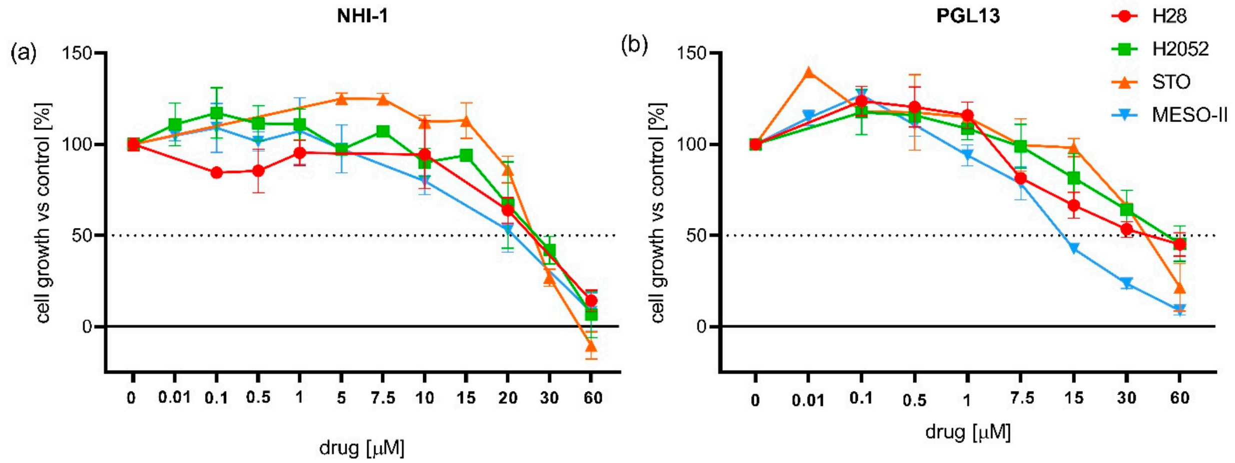

Supplementary Figure S1. Growth inhibition curves, showing the growth inhibition with NHI-1 (a) and PGL13 (b) in malignant mesothelioma cell lines after 72 h treatment in normoxia. Results are presented as means ± SEM; n=3.

Supplementary Table S1. Intracellular nucleotide concentrations in pleural (H28, H2052) and peritoneal (MESO-II, STO) mesothelioma cells after 24 h exposure to GLUT-1 (PGL13, PGL14) and LDH-A (NHI-1, NHI-2) inhibitors at their IC_{50} concentrations.

	cell line	inhibitor	ATP	ADP	AMP	NAD^+	NADH
pleural MM	H28	control	10.6 ± 0.4	0.49 ± 0.03	0.37 ± 0.02	1.9 ± 0.1	0.005 ± 0.002
		PGL13	8.00 ± 0.01 ***	0.395 ± 0.003*	0.32 ± 0.01*	1.30 ± 0.01 ****	0.027 ± 0.004 **
		PGL14	10.3 ± 0.3	0.44 ± 0.02	0.34 ± 0.01	1.73 ± 0.04	0.04 ± 0.01
		NHI-1	10.7 ± 0.1	0.54 ± 0.02	0.33 ± 0.01	1.86 ± 0.01	0.031 ± 0.003 ***
		NHI-2	4.6 ± 0.3 ****	0.93 ± 0.03 ****	0.47 ± 0.02 ***	1.11 ± 0.03 ****	0.12 ± 0.01 ****
	H2052	control	36.6 ± 1.5	1.8 ± 0.2	0.72 ± 0.02	6.5 ± 0.2	0.9 ± 0.1
		PGL13	33.2 ± 0.8	2.07 ± 0.03	0.57 ± 0.01	5.2 ± 0.1****	0.89 ± 0.01
		PGL14	21.3 ± 1.1 ****	3.3 ± 0.4***	0.7 ± 0.1	2.3 ± 0.1 ****	0.68 ± 0.02
		NHI-1	8.2 ± 0.4 ****	0.85 ± 0.04 ****	0.26 ± 0.02 ****	1.4 ± 0.1 ****	0.6 ± 0.1 *
		NHI-2	8.3 ± 1.5 ****	0.9 ± 0.1 ****	0.19 ± 0.03 ****	1.3 ± 0.2 ****	0.6 ± 0.1 *
peritoneal MM	MESO-II	control	27.8 ± 0.3	2.0 ± 0.1	0.450 ± 0.004	4.69 ± 0.02	0.32 ± 0.03
		PGL13	27.8 ± 0.9	2.1 ± 0.1	0.41 ± 0.01	4.1 ± 0.1 *	0.28 ± 0.01
		PGL14	27.9 ± 1.1	2.3 ± 0.1	0.46 ± 0.02	4.4 ± 0.2	0.31 ± 0.02
		NHI-1	25.3 ± 0.2 *	2.1 ± 0.1	0.43 ± 0.01	4.1 ± 0.1 ***	0.33 ± 0.01
		NHI-2	21.0 ± 0.6 ****	1.99 ± 0.03	0.36 ± 0.01 **	3.1 ± 0.1 ****	0.23 ± 0.01 *
	STO	control	20.0 ± 1.0	1.4 ± 0.1	0.22 ± 0.02	3.4 ± 0.3	1.3 ± 0.1
		PGL13	14.6 ± 0.6 ***	1.23 ± 0.03	0.20 ± 0.01	2.2 ± 0.1 **	1.15 ± 0.01
		PGL14	1.05 ± 0.01 ****	1.1 ± 0.2	0.6 ± 0.1 ***	1.2 ± 0.1 ****	1.3 ± 0.2
		NHI-1	16.3 ± 0.8	1.06 ± 0.02	0.182 ± 0.002	2.4 ± 0.2	1.1 ± 0.3
		NHI-2	17.7 ± 1.7	1.4 ± 0.1	0.24 ± 0.01	2.6 ± 0.3	1.38 ± 0.04

Results are presented as means ± SEM in nmol/mg protein; n=4, statistically different compared to control; one-way ANOVA * $p<0.05$; ** $p<0.01$, *** $p<0.005$, **** $p<0.0001$. LDH-A: lactate dehydrogenase A; GLUT-1: glucose transporter type 1; MM: malignant mesothelioma; IC_{50}: 50% cell growth inhibition concentration; ATP: adenosine triphosphate; ADP: adenosine diphosphate; AMP: adenosine monophosphate NADH/NAD^+: reduce/oxidized nicotinamide adenine dinucleotide.

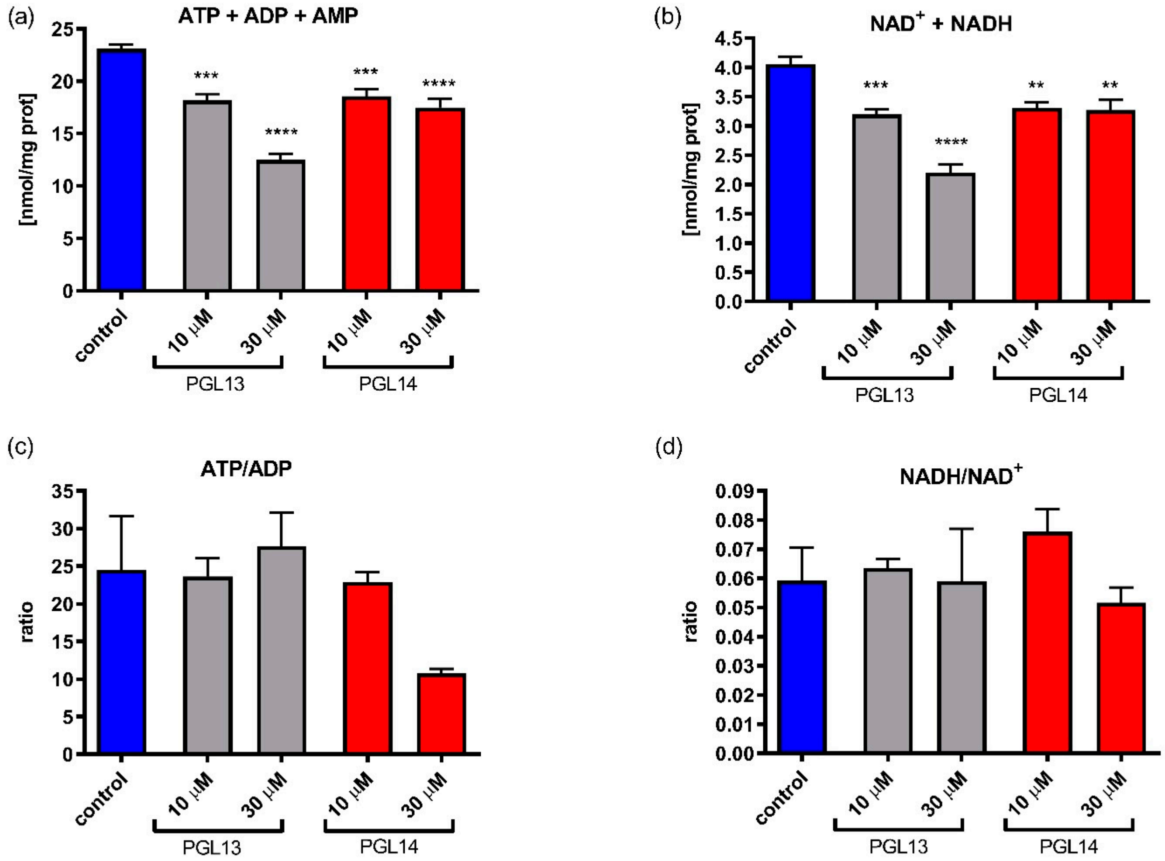

Supplementary Figure S2. Intracellular nucleotide concentrations (a, b) and nucleotide ratios (c, d) in a human microvascular endothelial cell line (HMEC-1) after 24 h exposure to GLUT-1 (PGL13, PGL14) inhibitors. Results are presented as means ± SEM; n=4, one-way ANOVA ** p<0.01, *** p<0.005, **** p<0.0001. GLUT-1: glucose transporter type 1; ATP: adenosine triphosphate; ADP: adenosine diphosphate; AMP: adenosine monophosphate NADH/NAD^{+}: reduce/oxidized nicotinamide adenine dinucleotide.

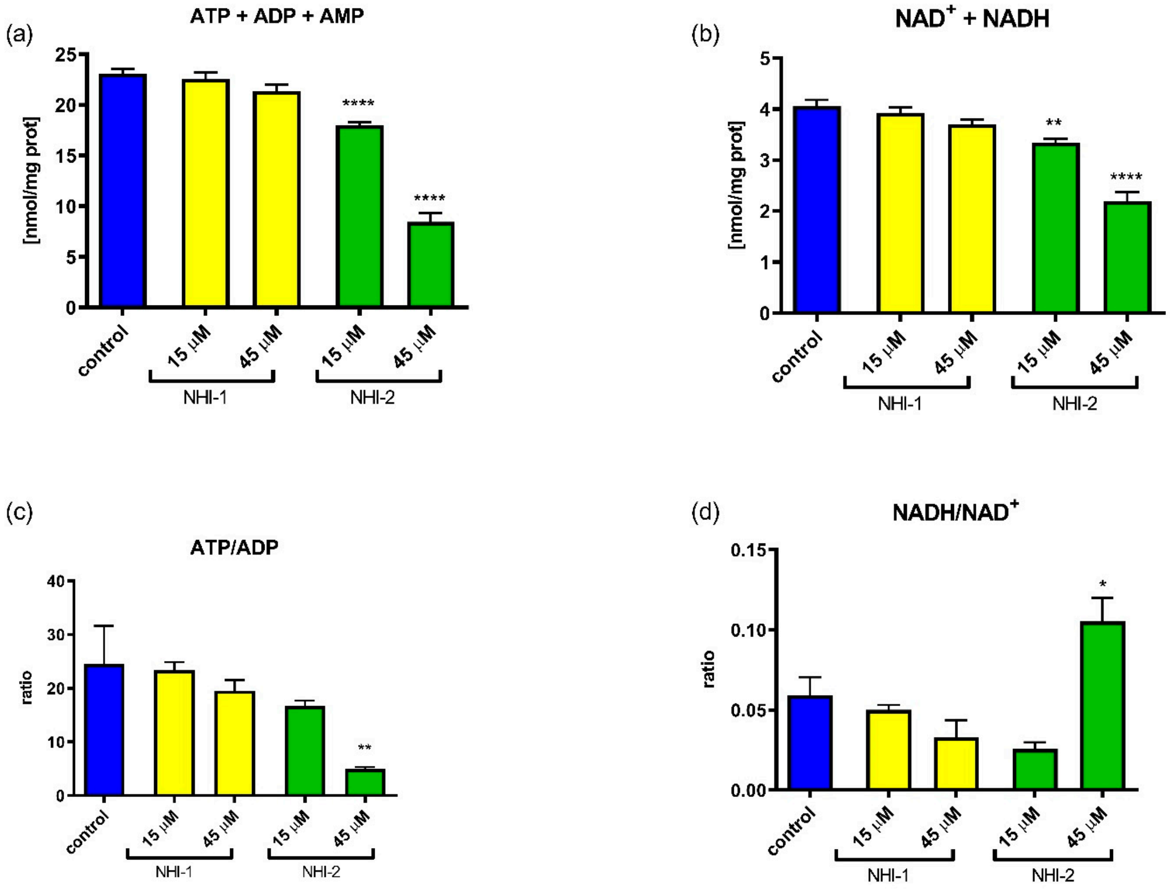

Supplementary Figure S3. Intracellular nucleotide concentrations (a, b) and nucleotide ratios (c, d) in a human microvascular endothelial cell line (HMEC-1) after 24 h incubation with LDH-A (NHI-1, NHI-2) inhibitors. Results are presented as means ± SEM; n=4, one-way ANOVA * $p<0.05$; ** $p<0.01$, **** $p<0.0001$. LDH-A: lactate dehydrogenase A; ATP: adenosine triphosphate; ADP: adenosine diphosphate; AMP: adenosine monophosphate NADH/NAD$^+$: reduce/oxidized nicotinamide adenine dinucleotide.
